# Predictors of poor blood pressure control among Iranian hypertensive patients

**DOI:** 10.1186/s13104-017-2971-4

**Published:** 2017-12-04

**Authors:** Leila Jahangiry, Jalileh Ghanbari, Mahdieh Abbasalizad Farhangi, Parvin Sarbakhsh, Koen Ponnet

**Affiliations:** 1 Health Education and Health Promotion Department, School of Public Health, Tabriz University of Medical Sciences, Tabriz, Iran; 2 Tabriz Health Services Management Research Center, Tabriz University of Medical Sciences, Tabriz, Iran; 3 Department of Community Nutrition, Faculty of Health and Nutrition, Tabriz University of Medical Sciences, Tabriz, Iran; 4 Epidemiology and Biostatistics Department, School of Public Health, Tabriz University of Medical Sciences, Tabriz, Iran; 5 Department of Communication Sciences, MICT-IMEC, Faculty of Political and Social Sciences, Ghent University, Ghent, Belgium; 6 Faculty of Social Sciences, University of Antwerp, Antwerp, Belgium

**Keywords:** Hypertension, Uncontrolled blood pressure, Patient, Cardiovascular diseases

## Abstract

**Objective:**

The aim of this study is to investigate factors associated with poor blood pressure (BP) control in older hypertensive patients living in Iran. Poorly controlled hypertension was defined as blood pressure greater than or equal to 140/90 mmHg. Multiple logistic regressions were performed to identify factors associated with poor BP control.

**Results:**

More than half of the patients (55.1%) had poor control of hypertension. Multivariate logistic regression analysis showed that being over 60 years of age (OR 1.67; 95% CI 1.18–2.37; *p* = .003), being widowed or divorced (OR 1.56; 95% CI 1.03–2.35; *p* = .035], smoking (OR 1.78; 95% CI 1.07–2.65; *p* = .01], BMI > 25 kg/m^2^ (OR 1.51 95% CI 1.05–2.78), having a waist circumference ≥ 90 cm (OR 1.7; 95% CI 1.2–2.42; *p* = .003], the use of calcium channel blockers (OR 2.69; 95% CI 1.26–5.72; *p* = .01], and the use of angiotensin converting enzyme inhibitors (OR 1.66; 95% CI 1.01–2.72; *p* = .044] contributed significantly to poor control of hypertension. Making a key BP control screening target (such as age over 60 and waist circumference of 90 cm or more) for cardiovascular specialists and other health care practitioners is needed for elderly patients at risk for poor BP control.

**Electronic supplementary material:**

The online version of this article (10.1186/s13104-017-2971-4) contains supplementary material, which is available to authorized users.

## Introduction

Hypertension is a common risk factor for cardiovascular disease and is a major health problem around the world. In addition, it is the most important single contributor to premature mortality [1–3]. It has been estimated that 22.1% of Iranian adults are hypertensive, and in most cases, their blood pressure is uncontrolled [4].

Poor control of blood pressure could augment cardiovascular risks, stroke, and myocardial infarction, which can be catastrophic to individuals and results in increasing costs for health care systems [5]. The role of systolic blood pressure (SBP) as an important predictor for cardiovascular risk has been demonstrated in several studies [6]. For instance, a recent study provided evidence that each 10 mmHg reduction in usual SBP is associated with a significant 11% reduction in the risk of myocardial infarction [7].

Because the need to control and to manage hypertension is well recognized, primary health care systems are on the front lines. They can provide screening programs for early detection, manage regular contacts with patients, apply preventive measures, and deliver consistent and persistent care for patients with hypertension [8]. However, it is important to identify the factors that influence BP control in hypertensive patients. Previous studies found that low adherence to medication use [9], an unhealthy lifestyle [10], competing priorities, and provider inertia [11] contribute to the poor control of BP. On the other hand, screening, diagnosis, treatment, and successful control of hypertension are difficult to catch, especially among older hypertensive patients. Adding another level of complexity to this public health problem, demographic variation and increasing prevalence of hypertension by age make this condition one of the major health policy challenges [12]. Approximately 50% of patients with hypertension drop out of health care within the first year of diagnosis, and only 25–34% of them comply with antihypertensive treatment and have controlled BP [13].

It is therefore important to identify factors that could affect BP control among older hypertensive patients. The aim of this study is to investigate factors that may influence poor BP control in older hypertensive patients and to identify patients’ characteristics associated with poor control of BP in order to improve the management and care of these patients.

## Main text

### Methods

This cross-sectional study was conducted in Takab, West Azerbaijan province, Iran. A total of 750 patients with a diagnosis of hypertension in primary health care centers were randomly recruited from August 2016 to February 2017. The inclusion criteria were: (1) having been diagnosed with hypertension in the previous 6 months, (2) being 50 years old or above, and (3) having a household health record in one of the Takab health care centers. Subjects who have diabetes and a cognitive disorder were not eligible.

Patients who had been referred to health care centers were randomly selected and contacted by telephone for eligibility. During the phone interview, eligible patients who were interested in participating in the study were asked to come to the health centers for a clinical assessment by a trained health care researcher. The response rate was 83.3% (750/900).

Data were collected by administering a questionnaire. The questions related to the participants’ sociodemographic characteristics, treatment for hypertension, medical history, and current cigarette smoking habits followed the guidelines provided by the American college of cardiology [14]. Furthermore, patients’ blood pressure (BP), waist circumference (WC), weight, and body mass index (BMI) were also recorded. BP was measured with a mercury sphygmomanometer, twice in the same arm, after the participant had been seated at rest for 10–15 min. The systolic and diastolic BP measurements were the mean of the two readings. WC was evaluated using a measuring tape to the nearest .1 cm. The weight of an individual dressed in light clothing without shoes was recorded each time using a calibrated scale to the nearest .1 kg. Height was measured without shoes using a stadiometer to the nearest .1 cm. BMI was measured by the individual’s weight in kilograms divided by the square of his/her height in meters [15].

Consistent with [16], uncontrolled hypertension was defined as systolic blood pressure ≥ 140 mmHg and/or diastolic blood pressure ≥ 90 mmHg for patients that currently taking antihypertensive medication. Antihypertensive treatment information was asked from patients and in the absence of patients information, it was derived from household health records (Additional file 1).

Statistical analyses were conducted using the STATA release 14.0 software (College Station, Texas, USA). Data were presented using frequencies (percentages) for categorical variables and means for numeric variables. Descriptive statistics were calculated to compare the characteristics of patients with controlled and uncontrolled hypertension. Between-group differences were calculated using χ^2^ or Student’s t tests. A series of t tests and multiple logistic regressions were performed to compute odds ratios (ORs) and their confidence intervals. In the univariate analyses, demographic and anthropometric factors were entered separately. p values below .05 were considered to be significant.

### Results

The sociodemographic and clinical characteristics of the hypertensive patients are shown in Table 1. The mean age of the 750 patients was 65.4 years, and 56.1% were women. More than half (55%, *n* = 413) of the patients had uncontrolled hypertension. There were significant marital status differences between patients with controlled and uncontrolled BP. Significant differences between patients with controlled and uncontrolled hypertension were found for BMI, weight, WC, years living with hypertension and comorbidities in hypertension (*p* < .05 for all).

**Table 1:** Descriptive characteristics of hypertensive patients

Parameters	Controlled BP	Uncontrolled BP	Total	p value
Categorical variables	N (%)	N (%)	N (%)	
Gender	337	413		.980
Male	148 (43.9)	181 (43.8)	329 (43.9)	
Female	189 (56.1)	232 (56.2)	421 (56.1)	
Marital status				.048
Married	275 (81.6)	304 (73.6)	579 (77.2)	
Single	4 (1.2)	2 (.48)	6 (.8)	
Widow	55 (16.3)	100 (24.2)	155 (20.66)	
Divorced	3 (.9)	7 (1.7)	10 (1.3)	
Education				.207
Illiterate	237 (81.6)	311 (75.3)	548 (73.1)	
Elementary school	90 (26.7)	97 (23.5)	187 (24.9)	
High school and university	6 (1.8)	3 (.7)	9 (1.2)	
Economic status				.671
Low	147 (43.6)	168 (40.6)	315 (42)	
Intermediate	139 (41.2)	177 (42.8)	316 (42.1)	
High	51 (15.2)	68 (16.4)	119 (15.8)	
Comorbidities of hypertension (yes)	72 (21.3)	130 (31.4)	202 (26.9)	.002
Mental	9 (12.5)	20 (15.4)	29 (14.3)	
Coronary Heart Disease	26 (31.7)	56 (68.3)	82 (40.6)	
Cancer	0	2 (1.5)	2 (.9)	
Respiratory diseases	12 (16.6)	21 (16.2)	33 (16.3)	
Urologic diseases	6 (8.3)	5 (3.8)	11 (5.4)	
Arthritis	12 (16.6)	17 (13.1)	29 (14.3)	
Thyroid disease	1 (1.3)	1 (.7)	2 (.9)	
Osteoporosis	2 (2.6)	2 (1.4)	4 (1.8)	
Eye diseases	1 (1.3)	3 (2.3)	4 (1.8)	
Gastritis	3	3 (2.3)	6 (2.9)	

Parameters	Controlled BP	Uncontrolled BP	Total	p value
Continuous variables	Mean (SD)	Mean (SD)	Mean (SD)	
Age (years)	64.7 (9.8)	65.9 (9.5)	65.4 (9.7)	.094
BMI (kg/m^2^)	26 (4.4)	27.4 (4.5)	26.8 (4.5)	< .0001
Weight (kg)	67.5 (12.2)	70.9 (12.5)	69.36 (12.5)	< .0001
WC	92.5 (12.3)	96.9 (11)	94.8 (12.6)	< .0001
SBP (mmHg)	125.9 (8.7)	147.1 (15.5)	137.6 (16.6)	< .0001
DBP (mmHg)	77.4 (6)	92.2 (8.8)	85.5 (10.6)	< .0001
Years living with hypertension	7.6 (4.4)	8.4 (4.4)	8.04 (4.5)	.018

*BMI* body mass index, *WC* waist circumference, *SBP* systolic blood pressure, *DBP* diastolic blood pressure

Table 2 provides information about patients’ medication use based on antihypertensive drug class. The most common medications used are angiotensin converting enzyme inhibitors (ACEIs, 37.9% of the patients), followed by angiotensin receptor blockers (ARBs, 14.9%), β-blockers (12.4%), diuretics (9.7%), and calcium channel blockers (CCBs, 3.9%). Almost all patients (99.9%) were receiving antihypertensive medication, and only 44% of the patients achieved controlled BP.

**Table 2:** Medication use among hypertensive patients based on antihypertensive drug class

Medication use	Controlled BP	Uncontrolled BP	Total
	337	413 (55.1)	750
CCB	10 (34.5)	19 (65.5)	29
ACEI	111 (39.1)	173 (60.9)	284
β-blockers	43 (46.2)	50 (53.8)	93
ARB	58 (51.8)	54 (48.2)	112
Diuretics	45 (61.6)	28 (38.4)	73
Diuretics + ARB	8 (28.6)	20 (71.4)	28
Diuretics + CCB	2 (20)	8 (80)	10
Diuretics + ARB + ACEI	4 (36.4)	7 (63.6)	11
β-blockers + ACEI	24 (66.7)	12 (33.3)	36
β-blockers + ARB	5 (31.3)	11 (68.8)	16
β-blockers + Diuretics	12 (63.2)	7 (36.8)	19
Diuretics + ACEI	13 (41.9)	18 (58.1)	31
ACEI + CCB	1 (14.3)	6 (85.7)	7

*ACEI* angiotensin converting enzyme inhibitor, *ARB* angiotensin receptor blocker, *CCB* calcium channel blockers

The results of the multivariate analyses of factors predicting poor BP control are shown in Table 3. Multivariate logistic regression analysis showed that the following factors were significantly associated with uncontrolled blood pressure control: being 60 years old or above (OR 1.67; 95% CI 1.18–2.37; *p* = .003), being widowed or divorced (OR 1.56; 95% CI 1.03–2.35; *p* = .035), smoking (OR 1.69; 95% CI 1.07–2.65; *p* = .023), BMI > 25 kg/m^2^ (OR 1.51 95% CI 1.05–2.78), having a waist circumference of 90 cm or more (OR 1.7; 95% CI 1.2–2.42; *p* = .003), using CCBs (OR 2.69; 95% CI 1.26–5.72; *p* = .01), and using ACEIs (OR 1.66; 95% CI 1.01–2.72; *p* = .044).

**Table 3:** Multivariate model predicting blood pressure control among patients with hypertension

	OR	95% CI	p value
Age in years (ref ≤ 60)			
> 60	1.67	1.18–2.37	.003
Gender (ref = female)			
Male	.79	.85–1.98	.226
Married status (ref = married)			
Single	.54	.084–3.5	.522
Widowed/divorced	1.56	1.03–2.35	.035
Smoking (yes)	1.78	1.07–2.65	.010
Comorbidity	1.32	.77–2.46	.329
Weight	1.01	.99–1.03	.21
BMI (ref ≤ 25 kg/m^2^)			
26–30 kg/m^2^	1.51	1.05–2.78	.025
> 30 kg/m^2^	1. 63	1.02–2.61	.040
Waist circumference in cm (ref ≤ 90)			
≥ 90	1.7	1.2–2.42	.003
Living with hypertension	1.24	.76–2.01	.39
Medication use			
CCB	2.69	1.26–5.72	.01
ACEI	1.66	1.01–2.72	.044
β-blockers	1.037	.62–1.7	.89
ARB	1.36	.84–2.21	.2
Diuretics	.94	.57–1.55	.82

*ACEI* angiotensin converting enzyme inhibitor, *ARB* angiotensin receptor blocker, *CCB* calcium channel blockers

### Discussion

The study presents an analysis of factors that may be associated with poor BP control in hypertensive patients in Takab, West Azerbaijan province, Iran. The findings from this study revealed that only 44.9% of the patients had adequate BP control [16]. A similar study from China indicated that nearly 95% of patients were receiving antihypertensive medication, and that only 16.7% of women and 19.1% of men had adequate BP control [17]. Although adequate control of BP is a cornerstone in stroke prevention and coronary events, our findings indicate that BP control cannot be achieved by more than half of the patients (55.1%). The rates of control of hypertensive patients in our study were higher than the 48% control rate in the United States of America that reported in a NHANES study [18]. Therefore, it is important to identify factors that influence poor BP control among hypertensive patients.

Results from the multivariate logistic regression analysis revealed that being older than 60 years, being widowed or divorced, smoking, having a waist circumference of 90 cm or more, the use of CCBs, and the use of ACEIs contribute to poor control of hypertension. The finding that being 60 years old or above is associated with higher odds of uncontrolled BP is consistent with previous studies [19]. One major reason for this poor management of BP is that the severity of hypertension increases markedly with advancing age [20]. Furthermore, because BP control is difficult in elderly patients and represents a management dilemma, it seems that categorizing hypertensive patients into ≥ 60 and < 60 years might be a key BP control screening target for cardiovascular specialists and other practitioners in health care settings. Ideally, older patients with hypertension should be followed up on every 3–6 months and continue to assess their adherence to medication use for BP control [21]. Also, more efforts, such as public health education and a blood pressure monitoring system, should be included for the older age group.

In this study, we found a significant association between poor BP control and being widowed or divorced, which is consistent with other studies [22, 23]. This finding may be related to the absence of partner support, which often results in lower health outcomes through health risk behaviors and stress [22]. Consistent with our results, Idler et al. [15] highlighted a strong protective effect of spouses in caregiving (both male and female) on survival for up to 5 years following surgery. This suggests that social spousal support, more so than lifestyle behaviors and medicine use, produces health benefits. Social support can benefit patients’ health by buffering stress, improving self-efficacy, and increasing healthy behavior [9, 24]. In fact, a meta-analysis study found that adherence was 27% higher when patients had practical support available to them. However, inconsistent with our results, there is also evidence that a spouse or partner does not reduce uncontrolled hypertension, suggesting that further research is needed regarding the role of spouses in the management of hypertension conditions [25].

Another factor that affected poor BP control is smoking. This result confirms that smoking acutely increases blood pressure due to an increase in both cardiac output and total peripheral vascular resistance [26]. Alternatively, smoker patients are more sedentary and have less healthy diets, both of which are factors that can directly raise BP and result in failure in BP control [27].

The relationship between waist circumference and hypertension has been clearly established [28]. In the present study, the likelihood of poor BP control was increased when the waist circumference was 90 cm or more (i.e., the cut-off point for the Iranian abdominal obesity definition) [29]. According to the findings of our study, an increase in waist circumference significantly increases the probability of poor BP control. This finding confirms that the impact of abdominal obesity on the control of hypertension is an important risk factor [30, 31].

All of the patients had been treated with antihypertensive drugs for an average of 8.04 years prior to study enrollment. Our findings suggest that poor control of blood pressure is also significantly associated with the use of CCBs and ACEIs, which is consistent with the findings of a study by Xu et al. [17]. However, some studies have shown improvements in the outcomes among patients with coronary heart disease using dihydropyridine CCBs [32, 33]. Although non-dihydropyridine CCBs are taken more often by uncontrolled than controlled hypertensive patients, they have marked negative inotropic effects [34]. Consistent with a previous study [34], our findings align with the efficacy of diuretics and renin-angiotensin system blockers, especially when they are taken in combination.

Hypertension surveillance could be used more frequently in daily practice in primary care to identify at-risk individuals. As such, it is important to make hypertensive patients and their families aware of the risk factors of uncontrolled BP. The results of the present study indicate the need for greater efforts at safe, effective strategies in order to achieve controlled BP among the oldest patients with hypertension. BP control may be related to factors beyond medication adherence, including lifestyle factors, social support, and the ability to navigate the health care system effectively, though this requires further study. In addition, the failure of antihypertensive medication to adequately control BP is determined by both the patients’ characteristics and factors related to the medication. The findings of this study indicate that treating elderly hypertensive patients will reduce the risk of cardiovascular events. There is evidence that hypertension treatment reduces the incidence of cognitive impairment and dementia in the elderly [35].

## Limitations

This study was cross-sectional, and could not determine causality. Another limitation was potential measurement error for the medication use. There wasn’t any information on actual adherence to high blood pressure medication use and treatment.

## Additional file

**Additional file 1.** Demographic questionnaire and personal information.

## Abbreviations

BP: blood pressure
sbp: systolic blood pressure
wc: waist circumference
bmi: body mass index
or: odds ratios
aceis: angiotensin converting enzyme inhibitors
arbs: angiotensin receptor blockers
ccbs: calcium channel blockers
