# Supplementary material for: Predictors of poor blood pressure control among Iranian hypertensive patients
Source: BMC Res Notes. 2017 Dec 4;10:668. doi: 10.1186/s13104-017-2971-4 (PMC5715656; doi:10.1186/s13104-017-2971-4)
Supplement: Supplementary file 1 — Additional file 1. Demographic questionnaire and personal information. [file 13104_2017_2971_MOESM1_ESM.docx]

**Demographic questionnaire**

**Personal information**

1. **Age:……………….years**
2. **Gender: Male □ female □**
3. **Marital status: Married □ Single □ Widowed/divorced □**
4. **Education:**

**a. Illiterate □ b. Elementary school □ c. High school□ d. University □**

1. **Currently smoking : No □ yes □**
2. **Do you have a disease? yes □ No□**

**- If your response is yes please state the types of your diseases............**

1. **Do you take medications? Yes □ No □**

**- If your response is yes please state the types of medications…………**

**8. History of hypertension……….**

**9. Type of medicine for hypertension……………..**

**10. Weight ……………..Kg**

**11. Height ………………..Cm**

**12. Waist circumference ………………Cm**

**13. Systolic blood pressure: 1.……………….2…………………3……………………..**

**14. Diastolic blood pressure: 1.……………….2…………………3……………………..**
